# Endovascular embolization for cases of ‘hidden’ pediatric cerebral arteriovenous malformations: A diagnostic & therapeutic challenge

**DOI:** 10.1016/j.ijscr.2021.106347

**Published:** 2021-08-31

**Authors:** Muhammad Arifin Parenrengi, Wihasto Suryaningtyas, Joandre Fauza

**Affiliations:** Department of Neurosurgery, Faculty of Medicine, Universitas Airlangga, Dr Soetomo Academic General Hospital, Indonesia

**Keywords:** Endovascular embolization, Pediatric arteriovenous malformations, Pediatric embolization, Transfemoral cerebral angiography

## Abstract

**Background:**

Ruptured arteriovenous malformations (AVM) hold a larger proportion as the cause of spontaneous intracranial hemorrhage in children compared to those in adults. Although surgical excision still remains as the gold standard therapy for arteriovenous malformations, some smaller ones are reported to resolve from embolization alone. However, difficulty arises when small arteriovenous malformations are not detected on certain diagnostic modalities such as Computed Tomography Angiography (CTA), giving rise to false negatives, which may compromise appropriate management of patients. Endovascular embolization can be used as alternative options as diagnostic and therapy for invisible arteriovenous malformation in children. We report two cases of ruptured paediatrics arteriovenous malformations with a complication of hydrocephalus, managed with endovascular embolization and a cerebrospinal fluid diversionary procedure.

**Case description:**

We report 2 case in from Dr. Soetomo academic general hospital in 2021, the first case was a fully conscious 6-year-old-female child with sudden left-sided weakness and severe headache in January, and the second case a 9-year-old female came with decreased consciousness in May. Both had evidence of intracerebral hemorrhage, intraventricular hemorrhage, and hydrocephalus on head radiological examination, but no visible vascular malformations on Computed Tomography Angiography. The first patient was treated with extra ventricular drainage initially, while the second case was not. Transfemoral cerebral angiography revealed small arteriovenous malformations in both patients, and both had successful endovascular embolization afterwards. The first case was shunt-free, while the second case had her drainage switched to ventriculoperitoneal shunt right after the embolization procedure. Both patients recovered fully without complications and sequelae, and were discharged afterwards.

**Discussion:**

Both patients did not undergo surgical resection of the arteriovenous malformations; the first case only underwent endovascular embolization, while the second case underwent embolization and ventriculoperitoneal shunting. The cases described here help highlight the irreplaceable role of Transfemoral Cerebral Angiography as a gold standard for cases for arteriovenous malformations compared to other modalities, such as Computed Tomography Angiography (CTA). Smaller arteriovenous malformations in paediatrics are reported to achieve complete radiological resolution, and cerebrospinal fluid diversion in hydrocephalic cases are not always performed. Several factors to be considered include initial consciousness and severity of neurological deficit, which were taken into account in the management of our patients.

**Conclusion:**

Embolization procedures may be beneficial in some pediatric arteriovenous malformations, preferably in smaller ones that undetectable by angiography. Several factors such as the consciousness and neurological deficit upon initial presentation may help in the decision making of these cases.

## Highlights

• Smaller AVMs may resolve from embolization alone
• CTA failed to detect AVM, whereas TFCA succeeded in doing so
• Two patients underwent AVM only and achieved complete neurological & radiological resolution
• CSF diversionary procedures may not always be necessary in hydrocephalic patients of ruptured AVMs

## 1 Introduction [1]

Arteriovenous malformations (AVM) of the brain are vascular connections anomalies with occurring in approximately 10–18 per 100,000 people [2]. Arteriovenous malformations may or may not produce symptoms; some remain silent for a long period of time, or can lead to seizures or rupture resulting in severe hemorrhage and debilitating neurologic sequelae. In pediatric populations, ruptured AVM hold a larger proportion as the cause of spontaneous intracranial hemorrhage than in adults [3]. The percentage may reach around 30%–50% in pediatric, while in adults the number sits at around 1.4, - 2% [4]. Conservatively managed paediatrics arteriovenous malformations are reported to have an annual risk of rupture of 4.4%–5.5% [3]. This number is significantly reduced after treatment, and this justifies early intervention of pediatric intracranial arteriovenous malformations [4], [5].

Therapeutic modalities of pediatric intracranial arteriovenous malformations include; observation and medications; surgical resection, which is the remaining gold-standard therapy; endovascular ^63^embolization; radiosurgery; and their combinations [4], [5]. However, surgical resection alone may sometimes be insufficient in managing arteriovenous malformations of higher grades and those situated at eloquent structures [4], [5], [7]. To help ease surgical resection, several endovascular modalities such as embolization may be useful in reducing the flow of larger arteriovenous malformations [8]. This may alleviate the symptoms of patients and shrink the lesion to increase the efficacy of further therapeutic approach such as surgery or radiosurgery [3].

Although generally embolization alone is unlikely to cure pediatric AVM [6], it has been reported to result in radiological resolution on 10%–40% of AVM [9], [10]. This chance of success is greater in those of smaller AVM [6]. However, risks of complications are still present, reportedly to be as high as 26% [8]. These may be procedure (vessel perforation, hematoma) or material-related (embolized pulmonary artery leading to pulmonary edema, bronchospasm due to solvent dimethyl sulfoxide use) [6], [11], [12].

In addition, some other intracranial conditions may be present including hydrocephalus or the disruption of cerebrospinal fluid (CSF) flow, resulting in its accumulation and deterioration of the general condition [12]. The presence of hydrocephalus itself indicates increased risk of poor neurological outcome after intracerebral (ICH) or intraventricular hemorrhage (IVH) [1]. In conditions such as these, CSF diversionary procedures, such as external ventricular drainage (EVD) or shunts may be needed. However, data regarding its application in pediatric population is not as abundant as those in adults [13], [14], and further studies and reports are still needed to evaluate its role and efficacy.

To further elucidate the role of embolization as alternative options of treatment of pediatric arteriovenous malformations, we report two cases of ruptured pediatric AVM with a complication of hydrocephalus, managed with endovascular embolization and a CSF diversionary procedure.

## 2 Case description

### 2.1 First case

This case series is reported in line with the PROCESS 2020 guideline [15]. The first case was of a 6-year-old female child referred to emergency room of Dr. Soetomo academic general hospital Surabaya Indonesia In January 2021, who presented with a sudden onset of left-sided weakness of the extremities 20 h prior to admission. The parents have given an informed consent for patient data publication. The patient also complaint of sudden and severe headache. There was nausea and vomiting occurring five times in total. The patient was fully conscious, and denied any history of trauma. There weren't any clinically significant medical conditions.

Physical examination revealed normal vital signs. Neurological examination with Brudzinski I revealed a nuchal rigidity and left-sided weakness of both the upper and lower extremities with positive pathological reflexes.

**Fig. 1:**
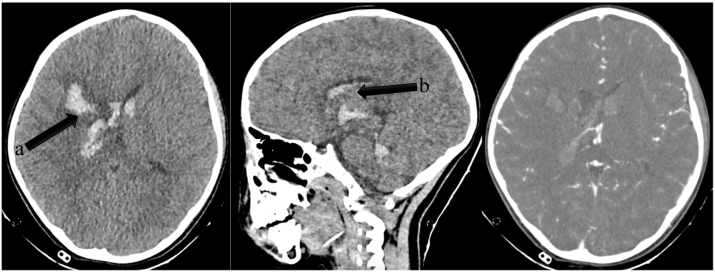
Head CT of the first case revealed a hyperdense lesion on the right frontoparietal lobe 4.6 × 2.7 × 4 cm in size and 24.8 cc in volume with an extension to the right lateral and third ventricle, and dilated ventricles, indicating the presence of right frontoparietal intracerebral (a) and intraventricular hematoma (b) along with non-communicating hydrocephalus. CT angiography (CTA) was performed but did not reveal any apparent vascular malformation.

Complete blood count, serum electrolyte and hemostatic profiles revealed normal results. Initial head computed tomography (CT) was performed, and revealed a hyperdense lesion on the right frontoparietal lobe 4.6 × 2.7 × 4 cm in size and 24.8 cc in volume with an extension to the right lateral and third ventricle, and dilated ventricles, indicating the presence of right frontoparietal intracerebral and intraventricular hematoma along with non-communicating hydrocephalus. Computed Tomography angiography (CTA) was performed but did not reveal any apparent vascular malformation (Fig. 1).

The patient was diagnosed with right frontoparietal intracerebral & intraventricular hematoma with a suspected cause of vascular malformation and non-communicating hydrocephalus. Due to the fully intact consciousness of the patient, the decision to treat the hydrocephalus with shunting was postponed and patient was planned to undergo strict and close observation while awaiting transfemoral cerebral angiography (TFCA) for diagnostic purposes.

**Fig. 2:**
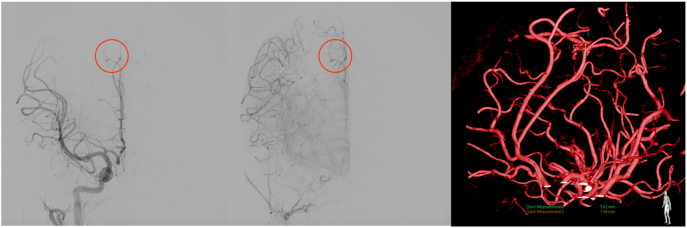
Angiography of the first case revealed the presence of vascular malformation (highlighted with red circles) in the right paracentral lobule with a fistula, feeding of an artery branching from posterior frontal artery and draining to the Rolandic vein 5.62 × 7.48 in size. (For interpretation of the references to colour in this figure legend, the reader is referred to the web version of this article.)

Angiography revealed the presence of vascular malformation in the right paracentral lobule with a fistula, feeding of an artery branching from posterior frontal artery and draining to the Rolandic vein 5.62 × 7.48 mm in size (Fig. 2). A diagnosis of ruptured pial arteriovenous malformation (AVM) of the right paracentral lobule was made. The patient was planned to undergo endovascular embolization, which was carried out on the second day after the trans femoral cerebral arteriography.

Endovascular embolization was performed using a general anesthesia and a 6 Fr. A branch of the right callosomarginal artery was embolized using Onyx 18 through the use of Marathon 1.5 Fr. (Minneapolis, Minnesota, USA) + Hybrid 0.008” detachable tip microcatheter (Irvine, California, USA) on the nidus from. is a detachable-tip microcatheter that was developed to reduce the risk of microcatheter entrapment during ethylene-vinyl alcohol copolymer (Onyx) embolizations. Post-embolization evaluation revealed complete obliteration of the nidus. The patient resolved into a full strength of the previously weakened extremities without any complications, and was discharged afterwards.

### 2.2 Second case

The second case was of a 9-year-old female child referred to Dr. Soetomo academic general hospital Surabaya Indonesia in May 2021, who presented with an abrupt decrease of consciousness 7 h prior to admission. The parents have given an informed consent for patient data publication. Her parents reported that she had previously complaint of severe, sudden headache, nausea, and slurred speech before falling into unconsciousness. There was also a left-sided weakness of the extremities. The patient denied any history of trauma. There weren't any clinically significant medical conditions.

Physical examination revealed an unstable hemodynamic status upon initial presentation. The patient was hypotensive, which was immediately successfully managed with fluid correction. Consciousness was decreased and lateralization was found indicated by weakness and hyperreflexia on the left extremities. Positive pathologic reflex was found on the lower left extremity.

**Fig. 3:**
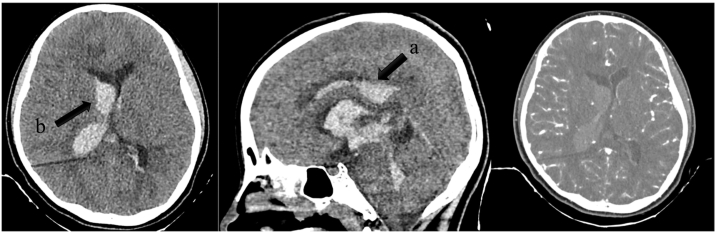
Head CT of the second case revealed the presence of a hyperdense lesion on the right parietal white matter 2.2 × 6.8 × 2.6 cm in size with a continuation to and filling the right and left lateral, third, and fourth ventricles (a), along with dilation indicating intracerebral & intraventricular hematoma with non-communicating hydrocephalus (b). CTA did not reveal any apparent vascular malformation.

There result for laboratory examination was leukocytosis, but otherwise normal laboratory results. Initial head Computed Tomography revealed the presence of a hyperdense lesion on the right parietal white matter 2.2 × 6.8 × 2.6 cm in size with a continuation to and filling the right and left lateral, third, and fourth ventricles, along with dilation indicating intracerebral & intraventricular hematoma with non-communicating hydrocephalus. Due to the uncommon presentation of pediatric intracerebral hemorrhage, a Computed Tomography Angiography was performed but no apparent vascular malformation was detected (Fig. 3).

**Fig. 4:**
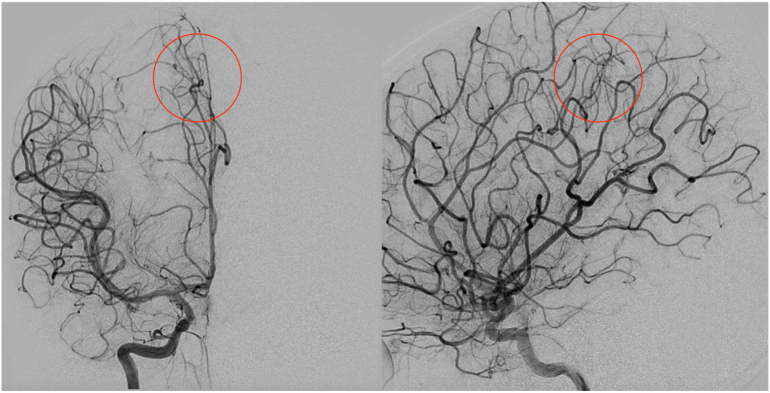
Angiography of the second case revealed a vascular malformation (highlighted in red circles) in the right parietal lobe with a feeding artery from the right pericallosal artery, branching of the right anterior cerebral artery. There was a nidus with a size of 4.86 × 2.43 mm in size, draining to the internal cerebral vein. (For interpretation of the references to colour in this figure legend, the reader is referred to the web version of this article.)

The patient was diagnosed with right parietal intracerebral & intraventricular hematoma with a suspected cause of vascular malformation and non-communicating hydrocephalus. Intravenous mannitol was administered. The decision to perform an extraventricular drainage was carried out on the left Kocher point, revealing an initial pressure of 25 cmH_2_O and clear reddish cerebrospinal fluid. The patient improved, and was planned to undergo transfemoral cerebral angiography (TFCA) for diagnostic purposes. Angiography revealed a vascular malformation in the right parietal lobe with a feeding artery from the right pericallosal artery, branching of the right anterior cerebral artery. There was a nidus with a size of 4.86 × 2.43 mm in size, draining to the internal cerebral vein (Fig. 4). Patient was diagnosed with a right parafalcine arteriovenous malformations, and the decision to perform embolization using onyx was made. Between the time of trans to embolization, an extraventricular drainage dependence test was performed and the patient was deemed ineligible for drainage removal. Thus, a ventriculoperitoneal shunt was also planned.

Endovascular embolization was performed using a general anesthesia and a 5 Fr. vertebral fermoral sheath. The right pericallosal artery was embolized using ONYX 18 + SQUID 12 through the use of a Sonic 1.5 (Montmorency, France) microcatheter detachable-tip from. Post-embolization evaluation was performed, and the AVM was no longer detected. There was a distal right internal carotid artery (ICA) and A1 segment of the anterior cerebral artery (ACA) vasospasm post-embolization. The contralateral ICA was evaluated and a sufficient collateral vascularization to the right ACA territory was detected. Ventriculoperitoneal shunting at medium pressure was performed right after the embolization procedure, without changing the entry point of the previously carried out EVD. The patient returned to full consciousness and extremity strength without any complications, and was discharged afterwards.

## 3 Discussion

Pediatric arteriovenous malformations are more prevalent as the cause of spontaneous intracerebral hemorrhage in compared to those in adults [3]. The percentage may reach around 30%–50% in paediatrics, while in adults the number sits at around 1.4, – 2% [4]. The gold standard diagnostic modality for cases of arteriovenous malformation is conventional cerebral angiography [16]. Conservatively managed pediatric arteriovenous malformation are reported to have an annual risk of rupture of 4.4%–5.5% [3]. Therapeutic modalities of pediatric intracranial include; observation and medications; surgical resection, which is the remaining gold-standard therapy; endovascular embolization; radiosurgery; and their combinations [4], [5]. Although surgical resection still remains as the gold standard for AVM management, aside from its role in easing surgery, endovascular embolization has been reported to result in radiological resolution on 10%–40% [9], [10], especially in smaller ones [6].

Both of our cases underwent computed tomography angiography upon initial presentation, due to a suspicion of vascular anomaly in a pediatric intracranial hemorrhage. However, both cases did not show an apparent malformation. In addition to the rarity of intracerebral hemorrhage in pediatric patients, the absence of malformation warrants further investigation, and thus cerebral angiography was carried out. These similar occurrences in both cases highlights the irreplaceable role of conventional cerebral angiography, at least up until now [16]. Cerebral angiography is reported to be able to show clinically important aspects, including feeding arteries, location of nidus, draining veins, morphology, presence and location of possibly present aneurysms, venous varices, and vasculopathic stenotic segments of blood vessel, which are all an important aspect in the planning of therapy of AVM cases [17]. Moreover, less invasive, but also less accurate diagnostic modalities such as CTA may help clinicians in diagnosing silent, asymptomatic, and unruptured vascular malformations, even though they may have to be large enough to be evident on the radiological imaging [16]. Although AVM are the leading cause of nontraumatic ICH in ages less than 35 years old, clinicians should still meticulously assess for the possibility of trauma and other causes of spontaneous rupture, even without vascular malformations [16], [18].

In our series, both patients did not undergo surgical resection of the AVM; the first case only underwent endovascular embolization, while the second case underwent embolization and VP shunting. Since the AVM was completely obliterated, through embolization alone, these cases may help to establish further studies regarding the possibility of embolization in replacing surgery as the definitive therapy for AVM, at least in paediatrics and small AVM [6]. Pezeshkpour et al. (2020) analyzed the outcome of embolization only therapy in pediatric AVM through a systematic review and meta-analysis [19]. A total proportion of 54.1% (range, 4–100%) had complete obliteration of the AVM [19]. Early complications after embolization were seen in 12.5% of patients, while delayed complications in 22.9% and recurrent hemorrhages were seen in 18 (9.7%) after treatment, while the mortality rate was 1.1% (1/93) [19]. Although the number of complete obliteration is not as high as those treated with surgery only (79.4%; range, 10–100%) or radiosurgery only (66.8%; range, 0–100%), this analysis showed that embolization only may be sufficient in an appropriate proportion of patients with AVM. Moreover, the AVM in that study are mostly Spetzler-Martin grade (SMG) I–III, which may, although not necessarily, indicate smaller size compared to those of grade IV & V [20]. Previous reports stated that children tend to have smaller nidi [7], [21], an important factor in the determination of success of embolization only for AVM therapy [6]. This could mean that endovascular embolization only may be more appropriate in most children, although personalized indications and therapeutic decisions should be made for every one of each patients. This is in accordance with the cases presented, in which the maximum lengths of the nidi are less than 3 cm, scoring only 1 on the SGM [20]. Although this study reported of appropriately high number of success through embolization, van Beijnum et al. (2011), in their systematic review and meta-analysis, noted less obliteration in both stereotactic radiosurgery and embolization with a success rate of 38% (range, 0–75%) in adult patients after stereotactic radiosurgery and 13% (range, 0–94%) after embolization [22].

Both patients had non-communicating hydrocephalus upon initial presentation, possibly due to the presence of intraventricular blood [12]. The presence of hydrocephalus could indicate an increased risk of poor neurological outcome after ICH or IVH [1], thus necessitating the use of a CSF diversionary device such as EVD or shunts. However, data regarding its application in pediatric population is not as abundant as those in adults [13], [14], and further studies and reports are still needed to evaluate its role and efficacy. A study by Stricker et al. (2020) analyzed the factors related to the need of EVD or VP shunt in 114 hydrocephalic children with ruptured cerebral AVM [1]. Their multivariate nominal logistic regression model identified that; low initial GCS, presence of hydrocephalus on initial CT scan, IVH, and higher modified Graeb Scale (mGS) score are strongly associated with subsequent need for EVD. In our case, the therapeutic approach of the hydrocephalus differed due to the difference, although not large, of initial GCS; the first patient had an initial GCS for 15, while the second had a decreased GCS of 13. This reasoning of our decision to do so is in accordance with the study by Stricker et al. (2020) where a point lower in initial GCS is associated with an adjusted OR of 0.74 (95% CI 0.98–1.37) of EVD success rate [1]. The same study also highlighted the important findings that EVD placement in the emergency setting does not increase the risks present in VP shunt insertion, making the proposition that indications for EVD placement after rupture of pediatric AVM can be open to a wider variety of cases to optimize the management of intracranial pressure [1]. Moreover, the severity of the initial neurological deficit also warrants CSF diversion, in which elevated ICP in the case of hydrocephalus is a considerable source of this morbidity [21]. The complications associated with CSF diversionary procedures also warrant the meticulous patient selection in hydrocephalic ruptured AVM cases. These include ICH caused by shunt insertion, infection, obstruction, abdominal pseudocyst, bowel perforation, and overdrainage leading to subdural hematoma [23].

## 4 Conclusion

Embolization procedures may be beneficial in some pediatric arteriovenous malformations. According to previous studies and the currently presented cases, smaller ones. Endovascular embolization can be used as treatment of undetected very small ruptured AVM in children. The use of CSF diversionary procedures may not always be necessary in hydrocephalic patients of ruptured AVM. Several factors such as the consciousness and neurological deficit upon initial presentation may help in the decision making of these cases.

## Disclosure of ethics

Written informed consent was obtained from the patient for publication of this case report and accompanying images. Medical data had been de-identified prior to publication to ensure patient's confidentiality.

## Funding

This study did not receive any funding or grant.

## Consent

Written informed consent was obtained from the patient for publication of this case report and accompanying images. A copy of the written consent is available for review by the Editor-in-Chief of this journal on request.

## Guarantor

Muhammad Arifin Parenrengi, M.D., PhD

## CRediT authorship contribution statement

To all persons who have made substantial contributions to the study, the author is showing his best gratitude to your endless support and attention for making this study happened.

## Declaration of competing interest

The authors declare that they have no known competing financial interests or personal relationships that could have appeared to influence the work reported in this paper.

## References

[1] Stricker S. (2020). Hydrocephalus in children with ruptured cerebral arteriovenous malformation. J. Neurosurg. Pediatr..

[2] Novakovic R.L., Lazzaro M.A., Castonguay A.C., Zaidat O.O. (2013). The diagnosis and management of brain arteriovenous malformations. Neurol. Clin..

[3] LoPresti M.A. (2020). Pediatric intracranial arteriovenous malformations: a single-center experience. J. Neurosurg. Pediatr..

[4] Darsaut T.E. (2011). Management of pediatric intracranial arteriovenous malformations: experience with multimodality therapy. Neurosurgery.

[5] Gross B.A., Storey A., Orbach D.B., Scott R.M., Smith E.R. (2015). Microsurgical treatment of arteriovenous malformations in pediatric patients: the Boston Children’s hospital experience. J. Neurosurg. Pediatr..

[6] El-Ghanem M. (2016). Arteriovenous malformations in the pediatric population: review of the existing literature. Interv. Neurol..

[7] Sorenson T.J., Brinjikji W., Bortolotti C., Kaufmann G., Lanzino G. (2018). Recurrent brain arteriovenous malformations (AVM): a systematic review. World Neurosurg..

[8] Soltanolkotabi M. (2013). Onyx embolization of intracranial arteriovenous malformations in pediatric patients. J. Neurosurg. Pediatr..

[9] Strozyk D., Nogueira R.G., Lavine S.D. (2009). Endovascular treatment of intracranial arteriovenous malformation. Neurosurg. Clin. N. Am..

[10] Barr J.C., Ogilvy C.S. (2012). Selection of treatment modalities or observation of arteriovenous malformations. Neurosurg. Clin. N. Am..

[11] Murugesan C. (2008). Severe pulmonary oedema following therapeutic embolization with onyx for cerebral arteriovenous malformation. Neuroradiology.

[12] Herman T.E., Siegel M.J., Vachharajani A., Masand P., Cross D. (2007). Cerebral arteriovenous fistula to pulmonary artery onyx embolization. J. Perinatol..

[13] Murthy S.B. (2017). Outcomes after intracerebral hemorrhage from arteriovenous malformations. Neurology.

[14] Gross B.A., Rosalind Lai P.M., Du R. (2013). Hydrocephalus after arteriovenous malformation rupture. Neurosurg. Focus..

[15] Agha R.A. (2020). The PROCESS 2020 guideline: updating consensus preferred reporting of CasE series in surgery (PROCESS) guidelines. Int. J. Surg..

[16] Ajiboye N., Chalouhi N., Starke R.M., Zanaty M., Bell R. (2014). Cerebral arteriovenous malformations: evaluation and management. Sci. World J..

[17] Mossa-Basha M., Chen J., Gandhi D. (2012). Imaging of cerebral arteriovenous malformations and dural arteriovenous fistulas. Neurosurg. Clin. N. Am..

[18] Ruíz-Sandoval J.L., Cantú C., Barinagarrementeria F. (1999). Intracerebral hemorrhage in young people. Stroke.

[19] Pezeshkpour P. (2020). Treatment strategies and related outcomes for brain arteriovenous malformations in children: a systematic review and meta-analysis. Am. J. Roentgenol..

[20] Al-Edrus S.A., Suhaimi S.N., Noor Azman A.R., Latif A.Z., Sobri M. (2010). The Spetzler-Martin grading system and management of patients with intracranial arteriovenous malformation in a tertiary referral hospital. Malays. J. Med. Heal. Sci..

[21] Blauwblomme T. (2014). Long-term outcome of 106 consecutive pediatric ruptured brain arteriovenous malformations after combined treatment. Stroke.

[22] van Beijnum J. (2011). Treatment of brain arteriovenous malformations. JAMA.

[23] Paff M., Alexandru-Abrams D., Muhonen M., Loudon W. (2018). Ventriculoperitoneal shunt complications: a review. Interdiscip. Neurosurg. Adv. Tech. Case Manag..

